# Exploitation of Design-of-Experiment Approach for Design and Optimization of Fast-Disintegrating Tablets for Sublingual Delivery of Sildenafil Citrate with Enhanced Bioavailability Using Fluid-Bed Granulation Technique

**DOI:** 10.3390/pharmaceutics13060870

**Published:** 2021-06-12

**Authors:** Amer S. AlAli, Mohammed F. Aldawsari, Ahmed Alalaiwe, Bjad K. Almutairy, Ramadan Al-Shdefat, Ismail A. Walbi, Mohamed H. Fayed

**Affiliations:** 1 Department of Pharmaceutics, College of Pharmacy, Prince Sattam Bin Abdulaziz University, Al-kharj 11942, Saudi Arabia; moh.aldawsari@psau.edu.sa (M.F.A.); a.alalaiwe@psau.edu.sa (A.A.); b.almutairy@psau.edu.sa (B.K.A.); 2 Department of Pharmaceutical Sciences, Faculty of Pharmacy, Jadara University, Irbid 21110, Jordan; rshdefat@jadara.edu.jo; 3 Department Clinical Pharmacy, College of Pharmacy, Najran University, Najran 55461, Saudi Arabia; iawalbi@nu.edu.sa; 4 Department of Pharmaceutics and Industrial Pharmacy, Faculty of Pharmacy, Fayoum University, Fayoum 63514, Egypt

**Keywords:** sildenafil, sublingual tablets, quality by design, fluid-bed, oral bioavailability

## Abstract

Sildenafil citrate undergoes first-pass metabolism, resulting in poor oral bioavailability at 25–41% of the administered dose. This study aimed to design and optimize fast-disintegrating tablets for the sublingual delivery of sildenafil citrate to improve bioavailability and facilitate rapid onset of action. The design-of-experiment (DoE) approach using 3^2^ full factorial design was conducted to develop a new formulation of sildenafil fast-disintegrating sublingual tablets (FDSTs) using the fluid-bed granulation technique. The levels of partially pre-gelatinized starch (5–15%) and microcrystalline cellulose (10–60%) were selected as independent formulation variables. The prepared FDSTs were investigated for physical properties. Further, the optimum formulation was chosen for in vivo study in rabbits. Regression analysis showed that independent variables have a significant (*p* < 0.05) influence on critical attributes of FDSTs. The optimized formulation showed acceptable mechanical strength (friability < 1.0%) with very fast disintegration (14.561 ± 0.84 s) and dissolution (94.734 ± 2.76% after 15 min). Further, the optimized formulation demonstrated a significant increase (*p* < 0.01) in C_max_ and AUC_0_–∞ with short t_max_ compared to the market product (Viagra^®^). Based on these results, using the DoE approach, a high level of assurance was achieved for FDSTs’ product quality and performance.

## 1. Introduction

Erectile dysfunction (ED) is the consistent or recurrent failure to obtain and/or maintain a penile erection necessary for adequate sexual performance [1]. Globally, the prevalence of ED is rated to be roughly 322 million cases by 2025 [2]. A recent study indicated that, 52% of men between the ages of 40 and 70 years suffered from ED [3]. It causes deep negative influence on an individual’s social life and prosperity [1]. Sildenafil “phosphodiesterase-5 inhibitors” is indicated as the first line treatment of ED [4]. Presently, sildenafil is only administered by the oral route. However, oral administration of sildenafil has several drawbacks. The bioavailability and pharmacological response are significantly influenced by gastric empty and first-pass metabolism. The absolute bioavailability of sildenafil in humans following oral administration of 50 mg was 41%. Additionally, a late onset of action was observed. After oral dosing, the onset of action usually started after 30–45 min [5,6].

Nasal, transdermal, and sublingual delivery systems have been investigated as alternative administration routes of sildenafil to improve bioavailability, attain rapid onset of action, and reduce the side effects associated with food intake [7,8]. However, sublingual delivery system is preferred to avoid the drawbacks associated with nasal and transdermal delivery systems [7]. The merit of the sublingual system is that the drug can be absorbed directly into systemic circulation, bypassing the first-pass effect and thereby increasing the overall bioavailability. Besides, the thin non-keratinized sublingual mucosa and the ample blood supply at the sublingual region allow for optimum drug penetration, resulting in higher plasma drug concentration and faster onset of action [9]. Moreover, it is convenient for the elderly and patients who have difficulty swallowing [6,10]. Clinical study showed that, with sublingual sildenafil, the onset of erection could be efficiently shortened to 15.5 min and not affected by food ingestion [6]. Another clinical study reported that all ED patients participating in the study preferred sublingual administration of sildenafil because of its rapid onset, which was unrelated to meal intake, especially in case of unplanned sexual activity [11]. Sheu et al. reported that sublingual delivery systems comprising sildenafil are good potential alternatives to conventional oral dosage forms [7].

Tablets are patient-friendly delivery systems that achieve better patient compliance [12]. Rapid disintegration and dissolution are critical quality attributes (CQAs) of tablet formulations used for sublingual delivery [7]. As a result, a combination of orally fast-disintegrating tablets (OFDTs), which “quickly disintegrate on contact with saliva before being swallowed” [13] and sublingual delivery appears to be an attractive strategy for the delivery of sildenafil for ED patients. There are several technologies for the manufacturing of OFDTs, including freeze-drying, sublimation, direct compression, and wet granulation processes. Fluid-bed granulation was reported to be the most appropriate granulation technology for the development of OFDTs [14]. In the pharmaceutical industry, fluid-bed granulation is a well-established technique, wherein process stages are performed using a single piece of equipment [12]. Furthermore, granules produced by the fluid-bed process have a higher porosity and specific density, as well as better compressibility and rapid dissolution [15]. 

To achieve optimal sublingual delivery, formulation variables have to be considered, as variability in formulation may result in failure of product quality [7,16]. Quality by Design (QbD) is a systematic approach approved by the Food and Drug Administration (FDA) for the manufacturing of safe and high-quality products [16]. Additionally, QbD is a wide expression, which includes a predefined quality target product profile (QTPP) and physicochemical, pharmacological, and clinical considerations to obtain products with desired attributes that are effective and safe [17]. Furthermore, the design of experiments (DoE) is a critical element of the QbD to achieve a better understanding of the influence of process and formulation variables on CQAs of the developed product by revealing the relationship between the independent process and formulation variables, and dependent response parameters [18]. 

The aim of the present study was to develop a new formulation of fast-disintegrating sublingual tablets (FDSTs) of sildenafil citrate with enhanced bioavailability and rapid onset of action using the fluid-bed granulation technique and DoE approach. Additionally, this study aimed to understand the impact of formulation variables (i.e., superdisintegrant, binder, and additives) on the CQAs of the sildenafil FDSTs product to ensure pharmaceutical quality, efficacy of drug product and patient safety. Furthermore, this study examined the bioavailability of prepared FDSTs and compared it with that of conventional tablets after administration to rabbits. QTPP and CQAs for sildenafil FDSTs are listed in Table 1.

## 2. Materials and Methods

### 2.1. Materials

Sildenafil citrate and D-Mannitol, Mannogem^®^, were kindly supplied by JPI Co. (Riyadh, Saudi Arabia). Microcrystalline cellulose (MCC), Avicel PH 101^®^, was procured from FMC biopolymer (Cork, Ireland). Partially pre-gelatinized starch (PGS), Starch 1500^®^, was purchased from Colorcon (Dartford, UK). Sodium stearyl fumarate, PRUV^®^, was kindly donated by JRS pharma (Rosenberg, Germany). Citric acid monohydrate was purchased from Sigma-Aldrich (Darmstadt, Germany). All other chemicals were of an analytical grade. 

### 2.2. Experimental Design

Using the Design-Expert software (Version-11, State-ease, Inc., Minneapolis, MN, USA), a 3^2^ full factorial design was created to investigate the individual and combined effects of independent formulation variables on CQAs of FDSTs. In this design, two independent variables were investigated at three levels. As shown in Table 2, the independent formulation variables studied were PGS concentration (X_1_) and the MCC concentration (X_2_). Table 3 shows the complete matrix of the design as obtained by the software. The chosen dependent responses were the d50 (Y_1_), bulk density (Y_2_), granules flow (Y_3_), the breaking force (Y_4_), friability (Y_5_), disintegration time (Y_6_), and percent of drug release after 15 min (Y_7_). 

The obtained data from various tests were presented as mean ± standard deviation (SD). The ANOVA test was performed for statistical analysis of the data using Design-Expert 11 software. A *p* value ≤ 0.05 was considered statistically significant.

### 2.3. Preparation of Sildenafil FDSTs

Table 4 shows the formulations used for the preparation of sildenafil citrate FDSTs using a fluid-bed granulation technique at 700 g scale. To prepare the binder solution, the required quantity of PGS was suspended in 250 mL of deionized water. Initially, D-mannitol and microcrystalline cellulose were blended in the V-mixer (VB-3, Erweka, Apparatebau, Langen, Germany) for 5 min at 70 rpm. After that, the mixture was loaded into the fluid-bed granulator (Huttlin mycromix, BOSCH Packaging Technology, Schopfheim, Germany) and granulated by spraying the binder solution (fluidizing air velocity 50 m^3^/h, inlet air temperature 60 °C, and spraying rate 6 g/min). After spraying the binder solution, the inlet air temperature was raised to 75 °C to dry the wet granules. In the V-mixer, the dry granules, citric acid, and lubricant were blended for 3 and 2 min, respectively. The final blend was then compressed with a single punch eccentric tablet press (Erweka EP-1, Apparatebau, Langen, Germany) using 10 mm shallow concave tooling at a fixed compression pressure of 12 kN into 300 mg tablets. The prepared tablets were collected and stored in opaque containers with desiccants for further characterization.

### 2.4. Characterization of Fast-Disintegrating Granules

#### 2.4.1. Mean Granule Size (d50) 

The laser diffraction technique was used to determine the d_50_ using Mastersizer 2000 (Malvern Instruments, Worcestershire, UK) at 25 °C with an angle of detection of 90°. During the test, a dispersing air pressure of 0.1–0.2 bar and a vibration of 20% were used. Laser obscuration was maintained between 0.6% and 6%. 

#### 2.4.2. Bulk Density (ρb)

The bulk density of obtained granules was measured using the method mentioned in USP [19]. Briefly, 30 g of prepared granules (m) was carefully poured into a 100 mL graduated cylinder up to a specific volume (Vb). The ρb was measured using Equation (1). Measurement was carried out three times.

ρb = m/V_b_ (1)

#### 2.4.3. Flowability 

The angle of repose method was used to determine the flowability of prepared granules [19]. Briefly, granules were cautiously poured through a dry funnel kept at approximately 2 cm (H), onto a clean flat sheet of paper to form a conical heap. Equation (2) was used to calculate the angle of repose between the surface of the powder heap and the surface of paper sheet (D).

tan (α) = 2H/D(2)

### 2.5. Tablet Characterization 

#### 2.5.1. Content Uniformity (CU)

The CU of obtained tablets was evaluated according to the USP standards [19]. Briefly, ten tablets were individually crushed and dissolved in methanol and filtered through a 0.45 μm membrane filter. Sildenafil content was determined spectrophotometrically (Shimadzu, UV-1700, Kyoto, Japan) at λ_max_ of 290 nm [20]. The acceptance value (AV) was determined using Equation (3).

AV = (X − M) + KS(3)

where X represents the average drug content, S represents the standard deviation and K represents a constant that is either 2.4 for 10 dosage units or 2.0 for 30 dosage units. If 98.5% ≤ X ≤ 101.5%; M = X, if X < 98.5%; M = 98.5%, if X > 101.5%; M = 101.5%.

#### 2.5.2. Weight Variation and Thickness Uniformity

A weight variation test was performed by weighing twenty randomly selected tablets (*n* = 20) on an analytical balance (Mettler Toledo New Classic ML204/01, Columbus, OH, USA). Individual tablet weights were compared to measure average weights. A digital micrometer (Mitutoyo, Natoli Engineering Co., Inc. Saint Charles, MO, USA) was used to measure the thickness of ten tablets (*n* = 10) placed perpendicular to the diameter. 

#### 2.5.3. Breaking Force (BF)

The BF test was performed for ten randomly selected tablets (*n* = 10) using a tablet hardness tester (Pharma Test, Hainburg, Germany). 

#### 2.5.4. Friability 

This test was carried out according to the USP standards [19]. A total of twenty randomly selected tablets (*n* = 20) were dedusted, weighed (W_1_), and placed in the friabilator (Pharma Test, Hainburg, Germany), which was then rotated 100 times. After that, tablets were removed, dedusted, and weighed (W_2_). Tablet friability was determined as percentage loss of weight using Equation (4). ≤1.0% loss in weight was considered acceptable.

Friability = (W_1_ − W_2_)/W_1_ × 100 (4)

#### 2.5.5. In Vitro Disintegration Test 

A USP disintegration tester was used to determine the DT for six randomly selected tablets (*n* = 6). The disintegration medium was 900 mL of distilled water heated to 37 ± 0.5 °C. The time required for the tablet to fully disintegrate was recorded in seconds. 

#### 2.5.6. In Vitro Dissolution Test 

This test was done for six randomly selected tablets (*n* = 6) according to USP procedure using a USP apparatus type II method at a paddle speed of 50 rpm. The dissolution medium was 500 mL of simulated saliva fluid (pH 6.76) at a temperature of 37 ± 0.5 °C. Samples were withdrawn at time intervals of 2, 5, 8, 15, and 20 min. Sildenafil content was analyzed using a UV spectrophotometer set at 290 nm [7,20].

### 2.6. Pharmacokinetic Study in Rabbits

#### 2.6.1. Animal Experiment 

This study was conducted to compare the pharmacokinetics of optimized FDSTs of sildenafil with commercially available tablets (Viagra^®^). Rabbits were chosen as convenient animal models to evaluate delivery potential in clinical studies because the sublingual mucosa of human and rabbit is non-keratinized. Besides, delivery of the drug to the sublingual cavity of the rabbit provides an opportunity to correlate the mechanism of intraoral absorption in rabbits with that of humans [21]. The present study was approved by Research Ethics and Animal Care Committee (Approval number: BERC-009-02-20) at College of Pharmacy, Prince Sattam bin Abdulaziz University, Saudi Arabia. The study was conducted using a single dose, two-period randomized crossover design with a one-week washout period after the last sample. Twelve healthy adult male New Zealand white rabbits (weighing 2–2.5 kg) were chosen for the study and held at room temperature (25 ± 2 °C). Prior to the experiment, the rabbits were fasted for 12 h and then divided into two groups (six rabbits per group). The optimized tablet (equivalent to 50 mg) was administered sublingually to the first group of animals using small tweezers. To avoid swallowing, the rabbit’s head was kept upright for 30 s after the tablet was administered. The other group of animals received the market product (Viagra^®^, Pfizer, Egypt) via oral administration of crushed tablets suspended in distilled water using a catheter. The blood samples (1 mL) were drawn via a rabbit’s marginal ear vein, at 0 (pre-dose), 2, 5, 10, 20, 30, 45, 60, 75, 90, 120, and 240 min after administration. Blood samples were immediately transferred to heparinized glass tubes and centrifuged at 3000 rpm for 15 min to separate plasma. The samples were then transferred to Eppendorf tubes and stored at −30 °C for drug analysis. 

#### 2.6.2. Plasma Treatment and Drug Analysis 

Plasma samples (100 µL) were placed in glass tubes and 10 mL of internal standard (butyl paraben; 40 mM in a phosphate-buffered solution (500 mM KH_2_PO_4_, pH 6.0) and 200 mL of acetonitrile were added and vortexed for 30 s. The tubes were then centrifuged for 5 min at 104 rpm and 4 °C. An aliquot of the supernatant solution (170 µL) was analyzed by the HPLC method as described by Yi et al. (2014) with modifications [22]. 

#### 2.6.3. Pharmacokinetic Analysis

The maximum plasma drug concentration (C_max_, ng/mL) and the time to reach C_max_ (t_max_, h) could be obtained from the plasma concentration–time curves. The t_1/2_ (h) was calculated as 0.693/K. The area under the curve (AUC_0–∞_, ng.h/mL) was calculated using the linear Trapezoidal rule. The obtained pharmacokinetic parameters are presented as mean ± SD and statistically compared using the ANOVA test. A *p* ≤ 0.05 was considered statistically significant.

## 3. Results and Discussion

### 3.1. Selection of Excipients 

Tablet properties can be affected by the properties of the excipients used in its formulation. Therefore, the selection of excipients and their proportions is important, and is based on the properties of the drug, desired formulation, and the method of manufacture [23]. PGS was chosen as binder/disintegrant as it enhances the flowability and compressibility of prepared granules alongside improving the disintegration of the prepared tablets [24]. MCC was chosen as the diluent because of its binding property. Besides, compared to other brittle excipients, MCC is self-disintegrating and needs a limited amount of lubricant [25]. Mannitol was chosen as the diluent and sweetening agent, as it produces a cooling sensation post dissolving in the oral cavity. In addition, the high aqueous solubility of mannitol helps in tablet wetting. However, the amount of mannitol should be carefully adjusted to avoid possible competitive dissolution, as mannitol can compete with sildenafil citrate for dissolution in the small amount of saliva available in the sublingual area [26]. Sildenafil citrate has an unpleasant taste when dissolved in the sublingual area [22]. Therefore, citric acid was added as a taste-masking agent, which masked the bitter taste by more than 80%. Additionally, citric acid can increase the amount of saliva needed for tablet disintegration and dissolution by stimulating saliva secretion. Furthermore, citric acid has the ability to facilitate drug transmission through the sublingual mucosa [26]. Ultimately, sodium stearyl fumarate was chosen as a hydrophilic lubricant as it enhances the flow of poorly flowable blends, while having limited impact on tablet strength, disintegration, or dissolution [27]. 

### 3.2. Validation of Drug Analytical Method

Calibration curve of sildenafil citrate was linear (y = 0.0204x + 0.0051) and well correlated (R^2^ = 0.9998) within a range of 10.0–50.0 µg/mL for intra- and inter-day assay. The UV-spectrophotometric analytical method for the analysis of sildenafil citrate was validated to be suitable for the determination of sildenafil content. The mean percentage of recovery was found to be 100.4% and RSD was 0.6%. The proposed analytical method exhibited good reproducibility, intermediated precision and repeatability. RSD values were 1.2% (based on the assay in different laboratories), 0.4% (intra-day), and 0.8% (inter-day), indicating the high precision of the method.

### 3.3. Statistical and Diagnostic Analysis of the Models 

**Figure 1:**
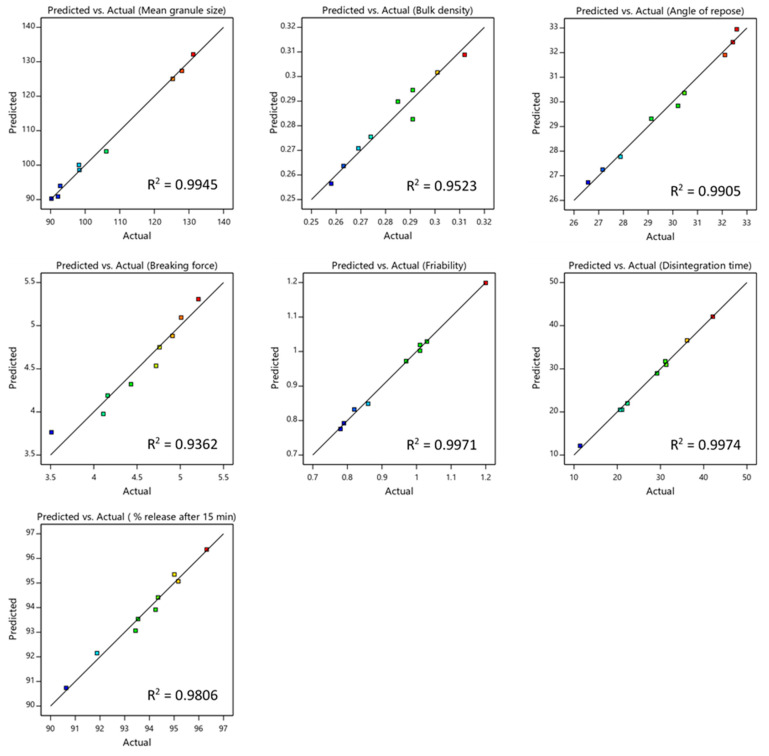
Linear correlation plot relating: mean granule size, bulk density, angle of repose, breaking force, friability, disintegration time and drug release after 15 min, between the predicted and the experimental values.

The results of the regression analysis of the proposed models are displayed in Table 5. It can be seen that models of all responses (Y_1_–Y_7_) showed a *p*-value < 0.05, which means that the model predictions were significant. Besides, the values of the actual model R^2^, adjusted R^2^, and predicted R^2^ were close to 1.0, which indicates a better model fit. Furthermore, Figure 1 displays a linear correlation between the actual and the predicted values for all responses that demonstrated a good model fit.

### 3.4. Granule Characterization

**Figure 2:**
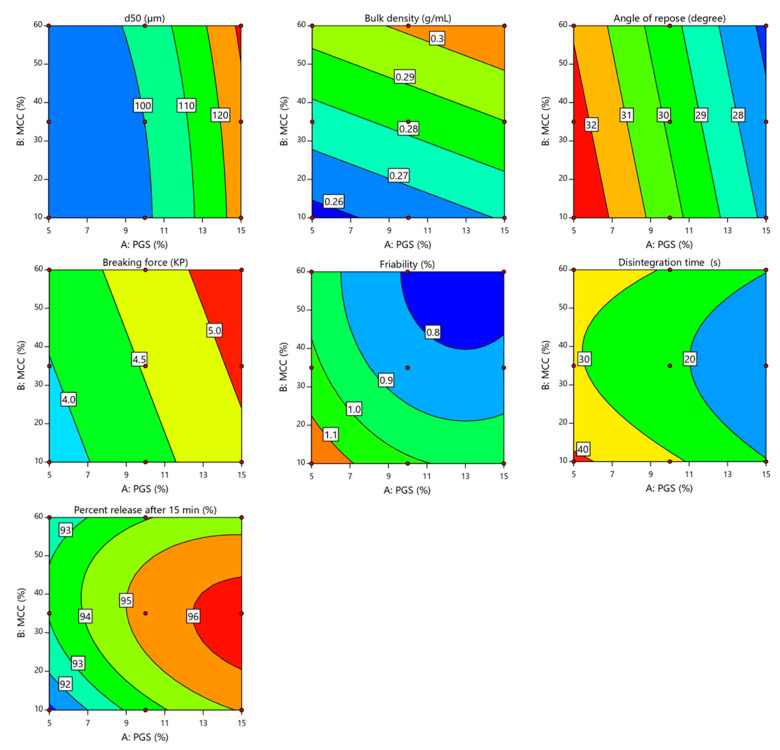
Contour plots showing the effect of the level of partially pre-gelatinized starch (X_1_) and the microcrystalline cellulose (X_2_) on mean granule size, bulk density, angle of repose, breaking force, friability, disintegration time and percent release after 15 min.

Table 6 depicts the physical properties of granules prepared using the fluid-bed granulation technique. It can be seen that increasing the partially pre-gelatinized starch (PGS) and microcrystalline cellulose (MCC) from 5.0% to 20.0% and from 10.0% to 60.0%, respectively, resulted in a significant increase in the average granule size (d50) from 90.25 ± 0.225 to 131.25 ± 0.522 µm. The results of the regression analysis are shown in Table 7. The results reveal that PGS and MCC had a significant impact on d50 (*p* = 0.0002 and *p* = 0.0462, respectively) in a positive direction, as indicated by the positive sign of the regression coefficient (+18.23 and +2.71, respectively). However, PGS level had the greatest impact on d50, as evidenced by the magnitudes of the sum of squares (1993.63 for PGS and 43.90 for MCC). Figure 2 showed an increase in the granule size with an increase in the PGS level due to the binding effect of the PGS [17]. 

As presented in Table 6, the bulk density of granules was increased from 0.258 ± 0.013 to 0.312 ± 0.016 gcm^−3^, due to an increase in the levels of PGS and MCC. ANOVA analysis, as shown in Table 7, showed that PGS and MCC levels had a significant effect on the bulk density of granules (*p* = 0.0083 and *p* < 0.0001, respectively). Additionally, the *p*-value demonstrated the pronounced effect of MCC. Moreover, PGS and MCC levels had a positive impact on granule bulk density with respect to the positive sign of the regression coefficient (+0.0072 and +0.0190, respectively). The contour plots (Figure 2) showed the dominant effect of MCC on granule density in a positive direction. This can be explained by the fact that, during the granulation process, MCC particles swell when they interact with water, followed by shrinking during the drying step. Increased intra-particle hydrogen bonding during the drying of MCC granules induces a marked increase in density and consequently a reduction in granule porosity [28].

The flowability of produced granules is shown in Table 6. It can be seen that the angle of repose values of prepared granules was lower than 33° (26.57 ± 0.316 to 32.59 ± 0.162). Consequently, they were classified as having good to excellent flowability (free flowing) according to USP standards for powder flow [19]. The results of the regression analysis, as depicted in Table 7, demonstrate that the levels of PGS and MCC had a significant impact (*p* < 0.0001 and *p* = 0.0062, respectively) on granule flowability. However, the effect of PGS was more pronounced on granule flowability considering the values of the sum of squares (40.15 for PGS and 1.63 for MCC). Additionally, PGS and MCC levels had a negative impact on the angle of repose values, as indicated by the negative sign of the regression coefficient (−2.59 and −0.5217, respectively). Figure 2 shows that the angle of repose was negatively affected by PGS and MCC levels, with PGS extending a dominant effect. The results suggest that granule flowability improved with an increase in the levels of PGS and MCC. Higher amounts of PGS and MCC could lead to an increase in granule size, which might result in an increase in granule flowability. The angle of repose values showed an excellent correlation with granule size (r^2^ = 0.9377). Mathematical models in coded terms generated by regression analysis are presented in the following equations.

d50 (µm) = 100.06 + 18.23 × X_1_ + 2.71 × X_2_ + 0.85 × X_1_ X_2_ + 00.906 × X_1_^2^ + 1.23 × X_2_^2^ (5)

Bulk density (gcm^−3^) = 0.2827 + 0.0072 × X_1_ + 0.0190 × X_2_ (6)

Angle of repose (degree) = 29.84 − 2.59 × X_1_ − 0.5217 × X_2_ (7)

### 3.5. Tablet Characterization

#### 3.5.1. Weight Variation, Thickness and Content Uniformity

The results of weight variation, thickness and sildenafil content in the prepared FDSTs are summarized in Table 8. For all formulations, the average tablet weight and thickness ranged from 299.52 ± 1.16 to 301.21 ± 1.43 mg and from 3.33 ± 0.013 to 3.32 ± 0.04 mm, respectively. All the prepared FDSTs displayed acceptable weight variation, as evidenced by the values of relative standard deviation of tablet weight that ranged from 1.16 to 1.43. The results indicate that the prepared granules have acceptable flow properties as previously discussed (Section 3.4). However, the observed slight variations in tablet mass could be attributed to a difference in the bulk density of granules [14]. On the other hand, sildenafil content ranged from 97.33 ± 1.94 to 100.53 ± 2.17% and relative standard deviations (RSD) were <6%. In addition, the acceptance value (AV) of sildenafil content was <15. This indicated that all prepared FDSTs showed acceptable content uniformity since they complied with the standards of United States Pharmacopeia (USP) for content uniformity [19].

#### 3.5.2. Breaking Force and Friability

It is important to estimate the breaking force and friability of the prepared tablets (i.e., tablet strength), as release of the drug in the patient’s body is significantly related to the strength of tablets [18]. As depicted in Table 8, all the prepared FDSTs showed a considerably low breaking force, which is preferred for rapid oral disintegrating tablets. The results of the ANOVA analysis, as shown in Table 9, reveal that PGS and MCC had a significant effect (*p* < 0.0001 and *p* < 0.0155, respectively) on tablet breaking force in a positive direction with respect to the sign of coefficient estimate (+0.5583 and +0.2133, respectively). However, PGS concentration has the most prominent effect on the breaking force of FDSTs, as evidenced by the magnitudes of the sum of squares (1.87 for PGS and 0.2731 for MCC). In addition, it was noted that the breaking force of prepared FDSTs was directly proportional to the concentration of PGS and MCC, as shown in Figure 2. This indicated that the breaking force of FDSTs was increased with an increase in the level of PGS and MCC. This observed effect is due to the better binding ability of PGS [29] as well as the excellent compactability of MCC at low pressure [25]. Therefore, the highest breaking force of FDSTs was obtained at a combination of high levels of PGS and MCC (Formula-9), as seen in the high right corner of the contour plot (Figure 2).

The regression analysis of the obtained data proved that the linear model was valid for tablet breaking force. Besides, the model significance was evidenced by the high *F*-value of 44.0 and a low p-value of 0.0003 with a correlation coefficient (R^2^) of 0.9362, thus assuring a good fit model. The equation that demonstrates the influence of tested variables on tablet breaking force is as follows:

Breaking force (KP) = 4.54 + 0.5583 × X_1_ + 0.2133 × X_2_ (8)

Friability is the second property related to the strength of the tablet. The main objective of the friability test was to estimate the ability of prepared tablets to resist abrasion during packaging and handling [30]. According to the USP criteria, prepared tablets exposed to the friability test should display weight loss <1% [19]. Besides, tested tablets should remain intact without any cracking or capping during the test. In some of the formulations with low levels of PGS and MCC, friable tablets were observed as shown in Table 8. Contrarily, formulations containing larger amounts of PGS and MCC passed the USP limit where friability was less than 1%. Further, all prepared tablets of these formulations showed no cracking, breaking or capping during tumbling in the friability tester. Regression analysis (Table 9) revealed that the PGS and MCC had a significant effect (*p* = 0.0003 and *p* = 0.00002, respectively) on the friability of prepared tablets in a negative direction based on the sign of the regression coefficient (−0.0983 and −0.1133, respectively). Additionally, PGS was the most influential variable affecting the friability of obtained tablets according to the magnitudes of the sum of squares (0.058 for PGS and 0.0771 for MCC). As displayed in Figure 2, an increase in the levels of PGS and MCC in the formulation resulted in a decrease in the friability of tablets. This suggested that the lowest friability value could be attained at a combination of high amounts of PGS and MCC, as depicted in the high right corner of the contour plot (Figure 2).

The regression analysis of the obtained data proved the validity of the quadratic model for testing tablet friability. However, the interaction effect seemed to be insignificant (*p* = 1.0) on tablet friability. Otherwise, the model significance was evidenced by the high *F*-value of 209.04 and low p-value of 0.0005 with a correlation coefficient (R^2^) of 0.9971, thus assuring a good fit model. The equation that demonstrates the influence of tested variables on the friability of tablets is as follows:

Friability (%) = 0.8489 − 0.0893 × X_1_ − 0.1133 × X_2_ − 0.0 × X_1_ X_2_ − 0.0817 × X_1_^2^ − 0.0567 × X_2_^2^ (9)

#### 3.5.3. In Vitro Disintegration Study

The disintegration of tablets is a critical attribute that needs to be optimized in a formulation of sublingual tablets [31]. Generally, orally disintegrating tablets have to disintegrate within seconds to a minute on the tongue and less than 30 s in the disintegration apparatus [13,32]. As shown in Table 8, the in vitro disintegration time (DT) for all prepared FDSTs was between 11.41 ± 0.52 and 42.11 ± 0.73 s. Besides, the DT of all formulations decreased when the amount of PGS in the tablets was increased from 5.0 to 15.0%. The results of the in vitro DT indicate that the formulations containing a high amount of PGS (F_4_–F_9_) show the most rapid disintegration. The rapid DT might be due to the ability of PGS to induce swelling as well as its low tendency to form a gelatinous mass on the peripheral area of the tablet, which acts as a barrier and prevents further water absorption by the tablet [24,29]. This finding agrees with Khafagy et al., who suggested that increasing PGS concentration reduced the DT of rapid orally disintegrating escitalopram tablets in a concentration-dependent manner [18]. It was also reported that PGS powders show sufficient swelling upon interacting with water, which results in a significant increase in tablet volume without blocking the porous structure or losing the wicking ability of the tablet. The concurrent increase in tablet volume and continuous capillary action of the disintegrant exert a massive force inside the tablet that led to rapid disintegration [33]. Moreover, the DT of prepared tablets slightly increased when the MCC load was increased to 60.0%, regardless of the concentration of PGS. Accordingly, MCC at 35.0% was considered the optimum proportion that resulted in the best DT. Therefore, the lowest DT of RDSTs was attained at a combination of a higher amount of PGS and intermediate amount of MCC.

Regression analysis (Table 9) demonstrated that the concentrations of PGS and MCC had a significant effect (*p* < 0.0001 and *p* = 0.0221, respectively) on the DT of the prepared tablet in a negative direction, as evidenced by the negative sign of the regression coefficients (−9.41 and −1.39, respectively). However, the magnitude of the sum of squares (530.72 for PGS and 11.51 for MCC) revealed that the rapid DT of RDSTs was strongly dependent on the concentration of PGS rather than MCC. Otherwise, the interaction effect between X_1_ and X_2_ had a significant impact (*p* = 0.0381) on DT in a positive direction (coefficient estimate = +1.38). As displayed in Figure 2, the DT was inversely proportional to the concentration of PGS and MCC with a rapid and sharp decrease in DT with an increase in the concentration of PGS and MCC from 5.0 to 15.0% and from 10.0 to 60.0%, respectively. Additionally, the fastest DT of 11.41 s was reported for F8 which contained the higher amount of PGS and an intermediate amount of MCC (15.0 and 35.0%, respectively) as shown in the middle of the contour plot. Furthermore, the regression analysis of the obtained data proved the validity of the quadratic model for the DT of RDSTs. The model significance was evidenced by the high *F*-value of 229.75 and low *p*-value of 0.0005 with a correlation coefficient (R^2^) of 0.9974 assuring a good fit model. The equation that demonstrates the influence of tested variables on the DT of tablets is as follows:

DT (s) = 21.99 − 9.41 × X_1_ − 1.39 × X_2_ + 1.38 × X_1_ X_2_ − 0.4317 × X_1_^2^ + 8.36 × X_2_^2^ (10)

#### 3.5.4. In Vitro Dissolution Study

**Figure 3:**
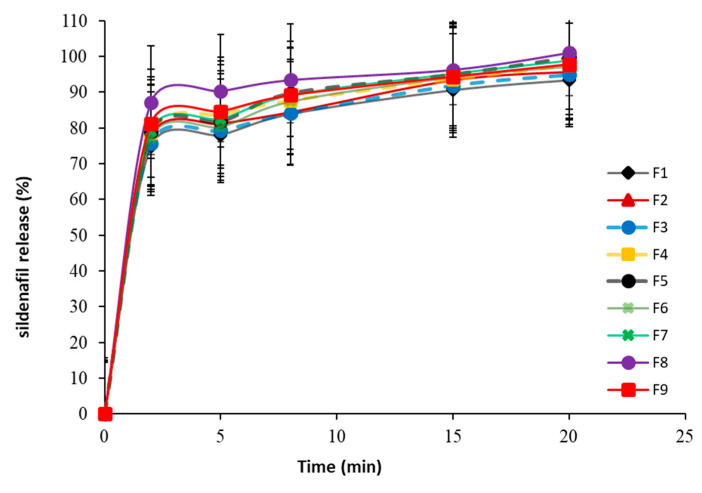
In vitro sildenafil release profiles from FDSTs based on 3^2^ full factorial design.

Dissolution of the tablet is an important parameter that is used to evaluate whether drugs are released in a defined and predictable manner [34]. Figure 3 shows the dissolution profiles of sildenafil citrate from prepared FDSTs at pH 6.76. The percentage of drug released after 15 min ranged from 90.63 ± 4.15 to 96.32 ± 4.01%. It was reported that the percentage of drug released from sublingual tablets must exceed 80.0% in 15 min [31]. All formulations showed an acceptable release profile, since they released more than 80.0% of sildenafil citrate in 15 min (Table 8). The results of the regression analysis (Table 9) reveal that PGS had a significant impact (*p* = 0.002) on sildenafil release after 15 min in a positive direction according to the positive sign of the regression coefficient (+1.65). Besides, the effect of PGS on sildenafil release was more pronounced than that of MCC, as evidenced by the p-value (*p* = 0.002 for PGS and *p* = 0.3231 for MCC) and magnitudes of the sum of squares (16.3 for PGS and 0.2166 for MCC). Figure 2 shows that the percent of sildenafil release after 15 min significantly increased with an increase in PGS. The rapid release of sildenafil could be explained as follows: initially, the dissolution medium rapidly penetrated the pores of prepared tablets that come in contact with PGS. The swelling of PGS was observed followed by the mechanical fragmentation of tablets into small agglomerates that resulted in the rapid release of sildenafil due to the availability of a higher surface area for dissolution [35]. Besides, fluid-bed produced low density and high porous granules that rapidly eroded, disintegrated, and rapidly released sildenafil from the tablet [12]. Furthermore, due to its higher aqueous solubility, the addition of mannitol to the formulation might facilitate the dissolution of prepared tablets [36].

The regression analysis of the obtained data proved the validity of the quadratic model for the sildenafil release from RDSTs. The significance of model was proved by the high *F*-value of 30.39 and a low *p*-value of 0.009 with a correlation coefficient (R^2^) of 0.9806, thus assuring a good fit model. The equation, which demonstrates the influence of tested variables on the release of sildenafil is as follows:

Release after 15 min (%) = 95.35 + 1.65 × X_1_ + 0.19 × X_2_ − 0.517 × X_1_ X_2_ − 0.635 × X_1_^2^ − 1.62 × X_2_^2^ (11)

#### 3.5.5. Optimization of Independent Variables

**Figure 4:**
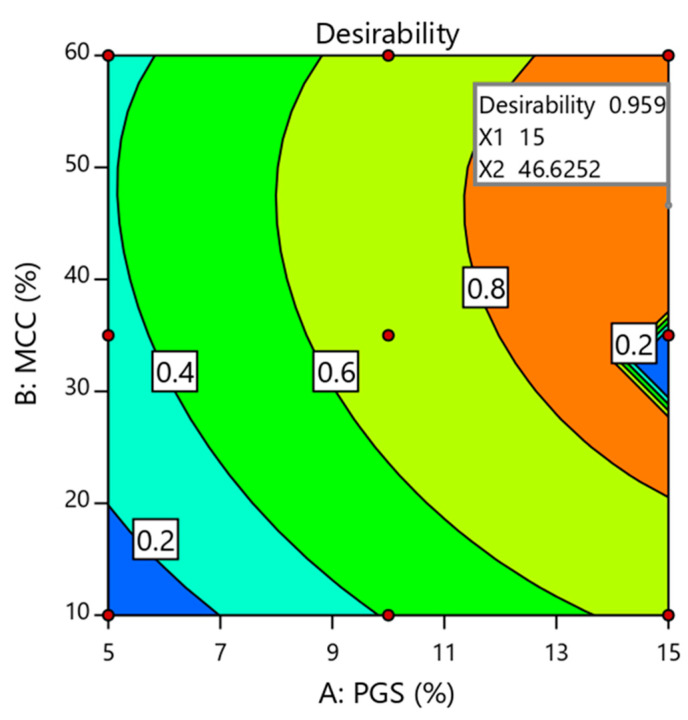
Optimization plot showing the influence of independent variables on overall desirability. X_1_ and X_2_ represent the concentration of partially pre-gelatinized starch and microcrystalline cellulose (respectively) in the optimized FDSTs’ formulation.

The main objective of the Design-Expert software is to find an optimized formula with the desired quality attributes [16]. In present study, the optimum values of independent variables were obtained using numerical optimization based on desirable conditions for all responses. In addition, the dissolution results did not consider in optimization process as all formulations satisfied the acceptance criteria. Thus, the criteria set for selection included attaining the maximum breaking force, minimum friability, and in vitro DT as depicted in Table 10. It was found that the formulation prepared at a combination of a high amount of PGS (15.00% *w*/*w*) and a moderate amount of MCC (46.62% *w*/*w*) achieved the required criteria with a higher desirability value of 0.959, as presented in Figure 4. Therefore, this formulation (optimized) was chosen for further in vivo study. For optimized formulation, the predicted and experimental values are listed in Table 11. There was an acceptable deviation (relative error < 5.0%) between the predicted and experimental values based on supposed models. This revealed the validity of the suggested design [37].

#### 3.5.6. Pharmacokinetic Assessment of Optimized Formulation

**Figure 5:**
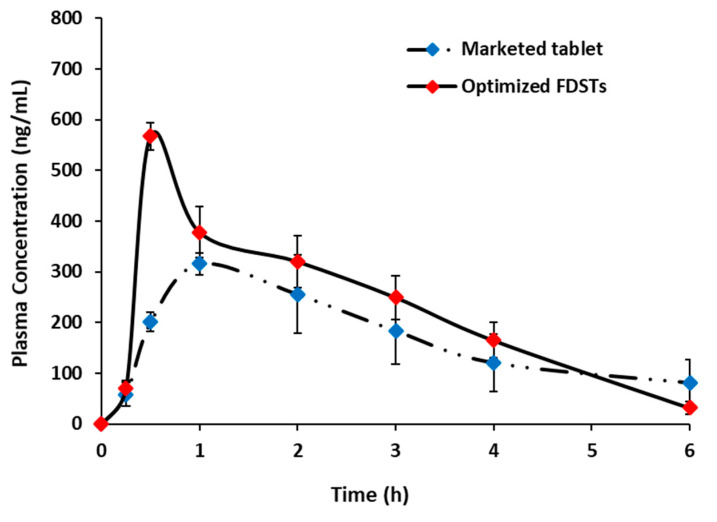
Plasma concentration–time profile of sildenafil after administration of optimized fast-disintegrating sublingual tablets (FDSTs) and market tablet product (Viagra^®^) to male Newzealand rabbits (*n* = 6, each value is the mean ± SD).

Figure 5 demonstrates the mean concentrations of sildenafil citrate in rabbits’ plasma versus the time following sublingual administration of the optimized formulation compared to the market oral product (Viagra^®^) as a reference. Pharmacokinetic parameters are listed in Table 12. Compared to the marketed oral tablet, the optimized formulation demonstrated a significantly (*p* < 0.01) higher C_max_ and AUC with a relative bioavailability of 160.52%. In addition, the optimized formulation showed a significantly (*p* < 0.01) shorter t_max_ than the oral marketed tablet. The results indicate that there was a difference in the rate and extent of absorption between the optimized formulation and market product [9]. The significant enhanced absorption of sildenafil from FDSTs could be attributed to: the thin non-keratinized sublingual mucosa and the ample blood supply at the sublingual area, which enables sildenafil to penetrate and achieve a high plasma concentration with rapid onset of action. The hepatic first-pass metabolism is bypassed through sublingual administration [7]. Moreover, rapid disintegration and release of sildenafil in the oral cavity is likely to enable pre-gastric absorption from the oral cavity, pharynx, and esophagus that can potentially enhance the bioavailability and help attain a higher C_max_ and a shorter t_max_ [9,38]. Although administration of FDSTs improved the oral bioavailability of sildenafil, some pharmacokinetic parameters differed from those previously reported. The t_max_ and C_max_ observed in the present study were shorter and higher than those reported for the sublingual administration of sildenafil tablets [7]. On the other hand, the present results coincide with the results reported by Hosny et al. [39]. They found that sublingual tablets of sildenafil citrate-PVP K30 co-precipitate (1:2 drug to polymer ratio) had better bioavailability (1.68-fold) than the conventional market tablet. However, in the present study, FDSTs demonstrated a notably higher C_max_ than the one reported by Hosny et al. due to the ultrafast disintegration and rapid dissolution of sildenafil citrate. Based on these results, the present study proves that fluid-bed granulation is an efficient technology for the improvement of dissolution rate, thereby leading to an improvement in the bioavailability of sildenafil citrate, without the use of other formulation strategies to enhance dissolution properties. Further, FDSTs could be considered to be a promising delivery system for sildenafil citrate, with expected enhanced bioavailability, rapid onset of pharmacological effect and better patient compliance.

## 4. Conclusions

The fluid-bed granulation technique could be useful for the development of FDSTs that are applied for sublingual delivery. Regression analysis indicated that formulation variables extended a significant (*p* < 0.05) impact on CQAs of FDSTs. Thus, selection of suitable excipients is critical for the development of FDSTs. The optimized formulation demonstrated acceptable mechanical strength (friability < 1.0%) with significantly fast disintegration (14.561 ± 0.84 s) and dissolution (94.734 ± 2.76% after 15 min). Further, the optimized formulation demonstrated a significant increase (*p* < 0.01) in C_max_ and AUC_0–∞_ with a short t_max_ compared to the market product. FDSTs of sildenafil represent a safe and applicable delivery system to achieve rapid onset of pharmacological effect. Further, FDSTs offer a significant enhancement in bioavailability via the bypassing of hepatic first-pass metabolism for the management of ED. FDSTs have been developed as an alternative delivery system to conventional tablets to improve patient convenience and acceptability and provide better compliance. The results of the present study demonstrate that the use of the DoE approach as a part of QbD tools enhances product quality and encourages an understanding of the influence of independent variables on the quality attributes of FDSTs.

## Figures and Tables

**Table 1:** QTPP and CQAs of sildenafil fast-disintegrating sublingual tablets (FDSTs).

QTPP Element	Target	CQAs	Justification
Dosage form	Fast-disintegrating sublingual tablets	Breaking force	Hard enough
Appearance	Uncoated tablets	Friability	<1%
Strength	50 mg	Disintegration time	<30 s
Route of administration	Sublingual	Drug release	More than 80% in 15 min
Proposed indications	Erectile dysfunction	-	-
Dosage frequency	Immediately before sexual activity	-	-

**Table 2:** The selected levels of independent formulation variables used in DoE.

Coded Levels	PGS Levels (%)	MCC Levels (%)
−1	5	10
0	10	35
1	15	60

−1: factor at low level; 0: factor at medium level; 1: factor at high level.

**Table 3:** A full matrix of 3^2^ full factorial design for sildenafil FDSTs’ formulations.

Formula	PGS Levels (%)	MCC (%)
1	5	10
2	5	35
3	5	60
4	10	10
5	10	35
6	10	60
7	15	10
8	15	35
9	15	60

**Table 4:** The quantitative composition of sildenafil citrate FDSTs’ formulations.

Ingredients	F_1_	F_2_	F_3_	F_4_	F_5_	F_6_	F_7_	F_8_	F_9_
Sildenafil citrate	16.66	16.66	16.66	16.66	16.66	16.66	16.66	16.66	16.66
Partially pre-gelatinized starch	5	5	5	10	10	10	15	15	15
Micro crystalline cellulose	10	35	60	10	35	60	10	35	60
Sodium stearyl fumarate	1	1	1	1	1	1	1	1	1
D-mannitol up to	100	100	100	100	100	100	100	100	100

All ingredients added in% *w*/*w*.

**Table 5:** Model summary statistics of dependent responses.

Response	Model	*F*-Ratio	*p*-Value	R^2^	Adjusted R^2^	Predicted R^2^
Mean granule size (d50)	Quadratic	2.92	0.0014	0.9945	0.9853	0.9495
Bulk density	Linear	0.5821	0.0001	0.9523	0.9364	0.9118
Angle of repose	Linear	2.10	<0.0001	0.9905	0.9873	0.9774
Breaking force	Linear	0.6099	0.0003	0.9362	0.9149	0.8436
Friability	Quadratic	0.060	0.0005	0.9971	0.9924	0.9736
Disintegration time	Quadratic	4.020	0.0005	0.9974	0.9931	0.9703
Drug release at 15 min	Quadratic	0.5776	0.0090	0.9806	0.9484	0.8142

**Table 6:** Physical properties of granules prepared by the fluid-bed granulation technique.

Formula	Mean Granule Size (µm) (mean ± SD)	Bulk Density (gcm^−3^) (mean ± SD)	Angle of Repose (Degree) (mean ± SD)
1	90.25 ± 0.225	0.258 ± 0.013	32.59 ± 0.162
2	92.14 ± 0.255	0.274 ± 0.011	32.43 ± 0.335
3	92.78 ± 0.239	0.291 ± 0.008	32.11 ± 0.193
4	98.34 ± 0.157	0.263 ± 0.014	30.47 ± 0.106
5	98.21 ± 0.297	0.291 ± 0.006	30.21 ± 0.113
6	106.11 ± 0.413	0.301 ± 0.023	29.13 ± 0.241
7	125.32 ± 0.365	0.269 ± 0.012	27.88 ± 0.264
8	127.97 ± 0.421	0.285 ± 0.034	27.16 ± 0.375
9	131.25 ± 0.522	0.321 ± 0.016	26.57 ± 0.316

**Table 7:** Regression analysis of dependent responses of prepared granules.

Variables	Coefficient Estimate	Sum of Squares	Standard Error	*F*-Value	*p*-Value	95% CI Low	95% CI High
**Mean granule size “d_50_” (*Quadratic model*)**							
**Intercept**	100.06	**-**	1.50	**-**	**-**	95.28	104.85
**X_1_**	18.23	1993.63	0.8233	490.23	**0.0002**	15.61	20.85
**X_2_**	2.71	93.90	0.8233	10.8	**0.0462**	0.0850	5.33
**X_1_ X_2_**	0.850	2.89	1.01		**0.4611**	−2.36	4.06
**Bulk density (*Linear model*)**							
**Intercept**	0.2827	**-**	0.0015	**-**	**-**	0.2790	0.2864
**X_1_**	0.0072	0.0003	0.0019	14.93	**0.0083**	0.0026	0.0117
**X_2_**	0.0190	0.0022	0.0019	104.95	**<0.0001**	0.0145	0.0235
**Angle of repose (*Linear model*)**							
**Intercept**	29.84	**-**	0.0861	**-**	**-**	29.63	30.05
**X_1_**	−2.59	40.15	0.1055	601.57	**<0.0001**	−2.84	−2.33
**X_2_**	−0.5217	1.63	0.1055	24.47	**0.0026**	−0.7797	−0.2636

X_1_ and X_2_ are independent formulation variables, X_1_ X_2_ is the effect of interaction.

**Table 8:** Physical properties of prepared sildenafil FDSTs * (mean ± SD).

Formula	Weight (mg ± SD)	Thickness (mm ± SD)	CU ** (% ± SD)	Breaking Force (KP ± SD)	Friability (% ± SD)	DT *** (S ± SD)	% Release after 15 min (% ± SD)
1	299.52 ± 1.16	3.33 ± 0.013	98.96 ± 1.36	3.51 ± 0.85	1.30 ± 0.07	42.11 ± 0.73	90.63 ± 4.15
2	297.82 ± 1.40	3.32 ± 0.007	100.51 ± 0.96	4.11 ± 0.65	1.03 ± 0.13	31.34 ± 0.67	93.44 ± 2.17
3	300.91 ± 1.69	3.34 ± 0.004	97.33 ± 1.94	4.16 ± 0.76	0.97 ± 0.16	36.16 ± 0.92	91.88 ± 3.36
4	299.71 ± 1.38	3.36 ± 0.03	99.58 ± 1.52	4.43 ± 0.58	1.01 ± 0.05	31.12 ± 1.67	93.54 ± 3.55
5	299.81 ± 1.51	3.34 ± 0.005	98.69 ± 1.65	4.72 ± 0.88	0.86 ± 0.03	22.36 ± 0.42	95.01 ± 2.97
6	298.14 ± 1.60	3.35 ± 0.007	99.46 ± 1.92	4.76 ± 0.96	0.79 ± 0.06	29.21 ± 1.24	94.25 ± 3.28
7	298.35 ± 1.49	3.31 ± 0.008	100.53 ± 2.17	4.91 ± 0.79	1.01 ± 0.04	21.11 ± 1.13	95.17 ± 3.87
8	297.62 ± 1.28	3.33 ± 0.006	98.17 ± 2.14	5.01 ± 0.58	0.82 ± 0.03	11.41 ± 0.52	96.32 ± 4.01
9	301.21 ± 1.43	3.32 ± 0.04	99.22 ± 2.44	5.21 ± 0.73	0.78 ± 0.04	20.66 ± 0.79	94.35 ± 3.15

* FDSTs: Fast-disintegrating sublingual tablets, ** CU: content uniformity and *** DT: disintegration time.

**Table 9:** Regression analysis of sildenafil FDSTs’ dependent responses.

Variables	Coefficient Estimate	Sum of Squares	Standard Error	*F*-Value	*p*-Value	95% CI Low	95% CI High
**Breaking force (*Linear model*)**							
**Intercept**	4.54	**-**	0.0520	**-**	**-**	4.41	4.66
**X_1_**	0.5583	1.87	0.0637	76.79	**0.0001**	0.4024	0.7142
**X_2_**	0.2133	0.2731	0.0637	11.21	**0.0155**	0.0574	0.3692
**Friability (*Quadratic model*)**							
**Intercept**	0.8489	**-**	0.0091	**-**	**-**	0.8200	0.8778
**X_1_**	−0.0983	0.0580	0.0050	391.61	**<0.0003**	−0.1141	−0.0825
**X_2_**	−0.1133	0.0771	0.0050	520.20	**0.0002**	−0.1291	−0.0975
**Disintegration time (*Quadratic model*)**							
**Intercept**	21.99	**-**	0.5776	**-**	**-**	20.15	23.83
**X_1_**	−9.41	530.72	0.3164	883.72	**<0.0001**	−10.41	−8.40
**X_2_**	−1.39	11.51	0.3164	19.16	**0.0221**	−2.39	−0.3782
**X_1_ X_2_**	1.38	7.56	0.3875	12.59	**0.0381**	0.1419	2.61
**Percent release after 15 min (*Quadratic model*)**							
**Intercept**	95.35	**-**	0.2940	**-**	**-**	94.41	96.28
**X_1_**	1.65	16.30	0.1611	104.74	**0.0020**	1.14	2.16
**X_2_**	0.1900	0.2166	0.1611	1.39	**0.3231**	−32.26	0.7026
**X_1_ X_2_**	−0.5175	1.070	19.73	6.88	**0.0788**	−1.15	0.1102

X_1_ and X_2_ represent the independent formulation variables; X_1_ X_2_ is the effect of interaction.

**Table 10:** The constraints adopted for the optimization of process variables and determination of overall desirability.

Variables	Target	Range	Weight	Importance Co-Efficient
**In-put**				
PGS	In range	5–15%	1	-
MCC	In range	10–60%	1	-
**Out-put**				
Breaking force	Maximize	3.51–5.21 KP	1	+++
Friability	Minimize	0.78–1.2%	1	+++
Disintegration time	Minimize	11.41–42.11 s	1	+++

**Table 11:** The quantitative composition of the optimized formulation. Predicted and experimental values for all dependent responses of optimized formulation with their relative errors.

**Ingredients**		**% *w*/*w***	
Sildenafil citrate		16.66	
Partially pre-gelatinized starch		15.00	
Micro crystalline cellulose		46.62	
Sodium stearyl fumarate		1.00	
D-mannitol up to		100.00	
**Responses**	**Predicted Values**	**Experimental Values (Mean ± SD)**	**Relative Error (%)**
Breaking force (KP)	5.193	5.382 ± 1.63	−3.639
Friability (%)	0.791	0.753 ± 0.48	4.804
Disintegration time (s)	13.958	14.561 ± 0.84	−4.320
Percent release after 15 min (%)	95.857	94.734 ± 2.76	1.171

**Table 12:** Plasma pharmacokinetic parameters of optimized sildenafil FDSTs and conventional marketed product tablets (Viagra^®^), results presented as mean ± SD.

Pharmacokinetic Parameters	Sildenafil Marketed Product (*n* = 6)	Optimized Sildenafil FDSTs (*n* = 6)
C_max_ (ng/mL)	327.92 ± 29.18	567.38 ± 27.36 **
t_max_ (h)	1.33 ± 0.51	0.50 ± 0.00 **
K (h^−1^)	0.29 ± 0.11	0.72 ± 0.09 **
t_1/2_ (h)	2.57 ± 0.73	0.97 ± 0.11 **
AUC_0–6_ (ng.h/mL)	1232.63 ± 393.38	1978.69 ± 261.37 **
AUC_0–∞_ (ng.h/mL)	1572.97 ± 631.21	2024.74 ± 280.74 **
Relative bioavailability (F) based on AUC_0–6_	-	160.52%

** *p* < 0.01, significant difference compared with the corresponding “Market Tablet”.

## Data Availability

The datasets used and/or analysed during the current study are available from the corresponding author on reasonable request.
